# Measuring the quality of antenatal care in a context of high utilisation: evidence from Telangana, India

**DOI:** 10.1186/s12884-022-05200-1

**Published:** 2022-11-25

**Authors:** Emma Radovich, Monica Chaudhry, Loveday Penn-Kekana, K. Radha Krishnam Raju, Aparajita Mishra, Ramya Vallabhuni, Prashant Jarhyan, Sailesh Mohan, Dorairaj Prabhakaran, Oona M. R. Campbell, Clara Calvert

**Affiliations:** 1 Faculty of Epidemiology and Population Health, London School of Hygiene & Tropical Medicine, London, UK; 2 Public Health Foundation of India, Gurgaon, India; 3 Indian Institute of Public Health, Hyderabad (IIPHH), Hyderabad, India; 4 Centre for Global Health, Usher Institute, University of Edinburgh, Edinburgh, UK

**Keywords:** Antenatal care, India, Quality of care, Maternity care

## Abstract

**Background:**

Antenatal care coverage has dramatically increased in many low-and middle-income settings, including in the state of Telangana, India. However, there is increasing evidence of shortfalls in the quality of care women receive during their pregnancies. This study aims to examine dimensions of antenatal care quality in Telangana, India using four primary and secondary data sources.

**Methods:**

Data from two secondary statewide data sources (National Family Health Survey (NFHS-5), 2019–21; Health Management Information System (HMIS), 2019–20) and two primary data sources (a facility survey in 19 primary health centres and sub-centres in selected districts of Telangana; and observations of 36 antenatal care consultations at these facilities) were descriptively analysed.

**Results:**

NFHS-5 data showed about 73% of women in Telangana received all six assessed antenatal care components during pregnancy. HMIS data showed high coverage of antenatal care visits but differences in levels of screening, with high coverage of haemoglobin tests for anaemia but low coverage of testing for gestational diabetes and syphilis. The facility survey found missing equipment for several key antenatal care services. Antenatal care observations found blood pressure measurement and physical examinations had high coverage and were generally performed correctly. There were substantial deficiencies in symptom checking and communication between the woman and provider. Women were asked if they had any questions in 22% of consultations. Only one woman was asked about her mental health. Counselling of women on at least one of the ten items relating to birth preparedness and on at least one of six danger signs occurred in 58% and 36% of consultations, respectively.

**Conclusion:**

Despite high coverage of antenatal care services and some essential maternal and foetal assessments, substantial quality gaps remained, particularly in communication between healthcare providers and pregnant women and in availability of key services. Progress towards achieving high quality in both content and experience of antenatal care requires addressing service gaps and developing better measures to capture and improve women’s experiences of care.

**Supplementary Information:**

The online version contains supplementary material available at 10.1186/s12884-022-05200-1.

## Background

The last few decades have marked substantial successes in increased coverage of essential maternal and perinatal health services in low- and middle-income countries (LMICs). In particular, coverage of antenatal care (ANC) has risen dramatically, as measured by whether women had four or more ANC visits, which alongside skilled birth attendant coverage, is one of the most widely used summary measures of maternal health programme performance [1, 2]. There are concerns that the ANC 4 + visit indicator has focused on advances in mere contact, rather than the process and content of ANC, obscuring large gaps between coverage of services and the quality of care received [1, 3]. This coverage-quality gap has been blamed for the persistent burden of maternal and perinatal mortality and morbidity [4, 5].

Several studies have combined indicators of ANC contact with capture of the care components received in order to measure ‘effective coverage’ for pregnancy care [1]. These studies mostly rely on household surveys, such as the Demographic and Health Surveys, which use women’s self-reports of care components received during the most recent pregnancy that ended in a live birth. This includes some routine elements that should be done at each ANC visit (e.g., blood pressure (BP) measurement), but women are asked only if they received the component at least once. The studies found that ANC 4 + visits and coverage across selected components of care correlated relatively well, as fewer visits meant fewer opportunities to offer/obtain care components [1, 3]. But while some LMICs had high coverage and high content of ANC, many did not; perhaps more troubling, some had high coverage but poor content [1]. In India, for example, the National Family Health Survey 2015–16 found that 51.2% of women had at least four ANC visits [6]; further analysis, however, revealed that only 23.5% of all women received adequate ANC which was defined as care delivered by skilled health personnel, registration of pregnancy and first ANC visit within the first trimester, 4 + ANC visits and with appropriate content [7].

New measures are needed to understand the care pregnant women receive [1, 3, 8, 9]. In 2016, the World Health Organization (WHO) released new ANC guidelines, recommending an increase from four to eight or more ANC visits, emphasising person-centred care and well-being, and recognising the complexities of providing and monitoring quality ANC in diverse health systems [10]. The WHO conceptual framework for quality ANC highlights the multiple dimensions of quality, including content and women’s experience of care, and various inputs needed to deliver routine ANC, including equipment and competent healthcare providers [9]. Measures reflecting services received at least once during pregnancy, such as those assessed on household surveys, are limited in assessing whether women were adequately followed throughout their pregnancies [8]. Among screening components, such as for syphilis or anaemia, there are often no indicators for whether women screening positive received adequate treatment, resulting in data gaps for capturing maternal and foetal assessment and appropriate response [9]. Further, few studies consider women's experience of care. Examining these different dimensions and inputs is critical to creating a holistic picture of quality of ANC.

Rethinking ANC quality assessment is particularly helpful in settings with high coverage like Telangana, India where nearly all pregnant women access ANC [11]. In Telangana’s ANC programme, pregnant women are expected to receive frequent ANC visits, including two visits in the first trimester to a sub-centre and one to a primary health centre (PHC) to register the pregnancy, provide an obstetric history and receive preventive and screening interventions (such as haemoglobin and syphilis testing). If no risk factors are identified, then pregnant women should have monthly primary care level facility visits and two visits to a higher-level facility with a gynaecologist in the second and third trimester. Women identified with high-risk pregnancies have monthly primary care level visits alongside multiple visits with a gynaecologist at a higher-level facility. The National Family Health Survey 2015–16 showed that in Telangana, 75.0% of women with a live birth in the previous five years had 4 + ANC visits, and among those who received ANC, reporting of selected components, such as BP measured, was nearly universal [12]. Yet these coverage measures do not reveal whether pregnant women received the care components correctly, at the right time and frequency, and with an appropriate response.

This paper takes a multi-dimensional approach to examine quality of ANC in Telangana, India based on four different sources of data.

## Methods

### Data sources

Four quantitative data sources were analysed in this paper. Two comprised secondary analyses of statewide data: 1) the most recently available National Family Health Survey (NFHS-5) 2019–21 for Telangana; and 2) state health management information system (HMIS) data for 2019–20. Two were from primary data collection undertaken in 2019–20 in the context of formative research for a quality improvement intervention [13] in selected districts of Telangana: 3) a facility survey; and 4) observations of ANC consultations.

**Fig. 1:**
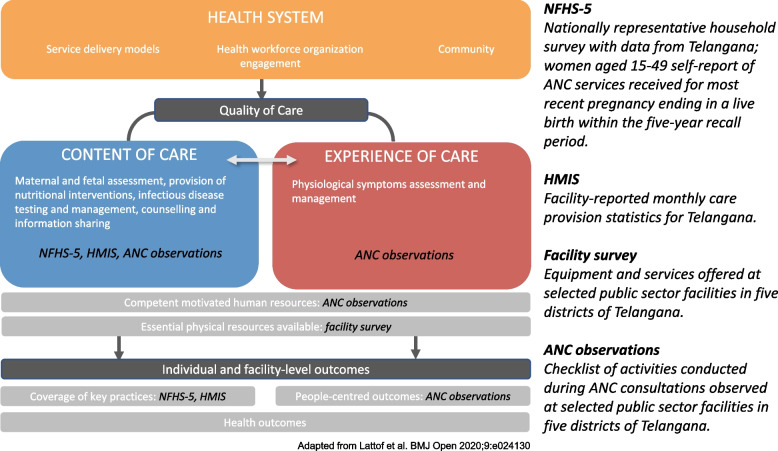
Four data sources included in this analysis mapped to the WHO framework for the quality of antenatal care. ANC = Antenatal Care

We adapted the WHO quality of care framework for ANC [9], situating the four data sources to show how we were capturing different components of the framework (Fig. 1).

### Data collection

#### NFHS-5 (2019–21)

The NFHS-5 was a nationally representative household survey using a multi-stage, cluster sampling design and providing national, state-level and district-level estimates of household and individual characteristics and reproductive health measures, amongst other topics. All women aged 15–49 in the selected households were eligible for interview. Data collection in Telangana was conducted from June to November 2019 [11]. Questions on ANC were asked of the pregnancy resulting in the most recent live birth in the five years before the survey.

#### HMIS

India’s Ministry of Health and Family Welfare (MoHFW) collects routine HMIS data primarily from public sector healthcare facilities, including monthly service delivery statistics [14]. In Telangana, HMIS data are digitally tracked by auxiliary nurse midwives at sub-centres and reported to their respective PHCs, which upload the aggregated data to the district level. Telangana aggregate HMIS data were obtained from the Commissionerate of Health and Family Welfare (CHFW) for ANC service delivery information for the period of April 2019 to March 2020.

#### Facility survey and ANC observations

Primary data collection was conducted in randomly-selected primary care level health facilities within five districts of Telangana (Medak, Rangareddy, Siddipet, Vikarabad and Yadadri Bhuvangiri). A list of public sector facilities was obtained from the CHFW for each district in Telangana. Facilities < 100 min driving time from the CHFW office in Hyderabad constituted the sampling frame. The sampling frame was then stratified by the facility level (sub-centres and PHCs) and two PHCs were selected at random from each of the five districts. Under these two PHCs, we randomly selected one associated sub-centre (total of two sub-centres in each district). After obtaining permission from the district health authorities, two trained research scientists visited the selected health facilities, and conducted the facility surveys and ANC observations.

A facility survey was conducted in 19 health facilities: 10 sub-centres and 9 PHCs. During data collection, one PHC selected from Yadadri Bhuvangiri was discovered to have been upgraded to a community health centre and was excluded from this study. The survey used a tailored ANC infrastructure assessment tool, adapted from the Service Provision Assessment facility inventory questionnaire [15]. The survey was administered using paper-based questionnaires by a trained researcher (KRKR) who obtained written informed consent and conducted interviews with the facility manager and the most knowledgeable staff person available for each health service area.

ANC observations were undertaken opportunistically at the selected study facilities; if a pregnant woman attended for ANC on the day the study team visited the facility, then the woman and the healthcare provider were asked to consent to have the ANC visit observed by a clinically-trained researcher (RV). ANC observations were guided by a checklist of routine activities based on relevant WHO and MoHFW of India guidelines and on a clinical observation tool previously used to assess routine childbirth care in Uttar Pradesh [16]. The ANC observation checklist covered activities that should be conducted either at the first or subsequent ANC consultations. The checklist was used to understand the process of care, how was it provided and how clinical notes and documentation of the ANC visit were captured in the client’s and facility records. The paper-based facility survey and ANC observation forms were double entered into Microsoft Access to ensure accuracy.

### Data analysis

#### NFHS-5 (2019–21)

All women aged 15–49 with a live birth in the survey’s five-year recall period living in Telangana were included in the analysis. For the pregnancy leading to the most recent live birth, we examined women’s self-report of the location(s) of their ANC, number of visits, timing (in months) of their first ANC visit, and the components of care received. These components included whether the woman was told about pregnancy complications, had her weight measured, abdomen examined, BP measured, and urine or blood samples taken during any of her ANC visits. We calculated the number of pregnant women who reported four or more ANC visits and those who reported eight or more ANC visits. Women who reported visiting any government health facility or government outreach programme (such as village clinic with auxiliary nurse midwives) were considered to have received ANC from a public sector facility. We additionally examined a subset of women who reported receiving ANC from a public PHC or sub-centre to facilitate comparisons to the other data sources. Less than 0.01% of women with a live birth were missing the number of ANC visits (*n* = 7); these were assumed to have had fewer than four visits. Two women were missing the timing of their first ANC visit and were assumed to have had their first visit after 4 months gestation. There was no other missing data in the analysis. The NFHS uses a multi-stage cluster sampling strategy, which we accounted for in statistical analyses.

#### HMIS

Due to likely underreporting from private sector facilities [14], we included only public sector service statistics in this analysis. All pregnant women registered for ANC seeking care from a public sector facility were included in the analysis. We extracted statewide service statistics from the 2019–20 report for total number of pregnant women registered for ANC, and amongst pregnant women registered we calculated the proportion who registered within the first trimester (up to 12 weeks gestation), received 4 + ANC visits, tested for blood sugar using oral glucose tolerance test (OGTT), received haemoglobin (Hb) tests four or more times in ANC, diagnosed with severe anaemia (Hb < 7), tested for syphilis, and diagnosed sero positive for syphilis. Amongst those with severe anaemia or syphilis, we also assessed the proportions who were treated.

#### Facility survey

We used survey data from 19 facilities, stratified by facility type, to look at two main domains: ANC basic equipment and ANC key services, reporting on the percentage of facilities that had an item within each domain, as well as the mean total score. For ANC basic equipment, we checked for the availability of a total of eight items of equipment required in delivery of routine ANC services: examination bed, measuring tape, height rod, examination light, BP measuring apparatus, stethoscope, fetoscope, and adult weighing scale. The functionality was also checked for five of the eight listed items (examination light, BP apparatus, stethoscope, fetoscope and adult weighing scale). For the ANC key services, or the infrastructure and processes to provide quality ANC, we evaluated whether ten key services were routinely offered and whether their associated equipment and commodities were available, functioning and, if relevant, had valid expiration dates. The services checked included iron and folic acid supplementation, tetanus toxoid vaccination, biochemical investigations (urine protein, blood/urine glucose, anaemia, and syphilis testing), routine measurements (weight, BP), and whether counselling was offered on eight core topics (minimum four visits, birth preparedness, planning transportation for delivery, family planning, breastfeeding, newborn care, postnatal care visits, healthy eating and physical activity). For a facility to be considered to offer an item within the ANC key services, they had to report that they provided the service and, where necessary, had the appropriate equiptment and supplies available.

#### ANC observations

We used data from 36 observations of ANC visits: 16 at sub-centres and 20 at PHCs. We assessed how well components of ANC were delivered by the healthcare providers by looking in detail at four domains: 1) respectful care (kind greeting, offered a seat, asked woman if she had any questions, discussed physical exam and washed hands with soap if undertaking a physical exam); 2) physical examination (BP, weight, fundal height, pallor, foetal heartbeat, oedema, foetal lie/presentation, pulse rate, respiratory rate and jaundice); 3) current symptom assessment (asked about decreased foetal movement, severe abdominal pain, persistent vomiting, severe difficulty breathing, vaginal bleeding, frequent painful urination, foul smelling vaginal discharge, swollen face or hands, headaches or blurred vision, woman’s mental health, palpitations, convulsions/loss of consciousness and fever); and 4) education (informed woman of pregnancy progress, counselled on danger signs, discussed nutrition and healthy eating, discussed next ANC visit details and counselled on birth preparedness).

Within each domain, key items from the observation checklist were identified by two clinically trained researchers. Items were tabulated to assess frequency of performance of routine activities. We excluded items not expected to be done at every visit. We restricted analysis for items that should be performed after 22 weeks gestation [10] to the observations of women who were at 22 weeks gestation or greater (assessment of fundal height, foetal heartbeat and foetal lie/presentation and asking about decreased foetal movement). To judge healthcare providers’ performance as an element of quality of care [9], we also examined nine indicators of good practice for measuring BP in the ANC observation tool: asked if patient had tea/coffee, back supported during measurement, feet rested, measurement taken on the left arm, arm rested, sleeve rolled-up, cuff band 1-2 cm above elbow, cuff at heart level, and deflation rate no more than 2–3 mm Hg/s [17–19]. We also reported the percentage of ANC consultations where the woman was tested, or referred for a test, for proteinuria, haemoglobin, blood/urine glucose and syphilis testing.

## Results

### NFHS-5

We included 5,429 women in Telangana whose most recent pregnancy ended in a live birth in the NFHS-5 (2019–20) analysis. Nearly all women had one or more ANC visits (99.3%), 70.5% had 4 + ANC visits (guideline during part of the survey’s recall period) and 20.7% of all women had 8 + ANC visits (the new WHO guideline) (Table 1). Nearly three out of four women received all six assessed content of care components during pregnancy (72.7%). Being told about potential pregnancy complications at any point during ANC was the lowest performed component (73.7% amongst all women). The remaining five care components were nearly universal (97–99%). Coverage of contact and content of care components received were similar amongst all women and amongst women who received care at public sector facilities.

**Table 1:** Number and percentage of ANC visits and components of care received for the most recent pregnancy that ended in a live birth in the five-year survey recall period among women living in Telangana, NFHS-5 (2019–20)

	***All women with a live birth in the five years before survey (n* = *5,429)***		***Women who attended for any ANC at a public sector facility (n* = *3,062)***		***Women who attended for any ANC at a public PHC or Sub-Centre (n* = *651)***	
	%	(95% CI)	%	95% CI	%	95% CI
***Contact***						
* 1* + *ANC visits*	99.3	(98.9 – 99.5)	-	-	-	-
* First visit at* < *4 months pregnant*	88.5	(87.4 – 89.5)	87.8	(86.2 – 89.3)	88.8	(85.3 – 91.6)
* 4* + *ANC visits*	70.5	(68.6 – 72.3)	71.8	(69.2 – 74.2)	76.7	(72.6 – 80.4)
* 8* + *ANC visits*	20.7	(19.0 – 22.5)	20.7	(18.6 – 23.1)	24.4	(20.3 – 29.1)
***Content***						
* Told about pregnancy complications*	73.7	(71.5 – 75.8)	74.7	(72.0 – 77.3)	77.1	(72.4 – 81.3)
* Weight measured*	99.1	(98.7 – 99.4)	99.8	(99.5 – 99.9)	99.6	(98.6 – 99.9)
* Abdomen examined*	97.3	(96.6 – 97.8)	97.9	(97.1 – 98.5)	98.9	(97.7 – 99.5)
* Blood pressure measured*	99.0	(98.6 – 99.3)	99.7	(99.4 – 99.8)	99.5	(98.5 – 99.8)
* Urine sample taken*	98.9	(98.5 – 99.2)	99.6	(99.3 – 99.8)	98.9	(97.7 – 99.5)
* Blood sample taken*	98.9	(98.4 – 99.2)	99.5	(99.1 – 99.8)	99.1	(97.9 – 99.6)
* All 6 above components received*	72.7	(70.5 – 74.8)	73.6	(70.8 – 76.1)	76.1	(71.3 – 80.3)

*CI*   Confidence Interval,  *ANC*   Antenatal Care

### HMIS

HMIS monthly reporting for public sector facilities for April 2019 to March 2020 showed high coverage of ANC visits and registration: 84.4% of registered pregnant women had 4 + visits and more than 70% registered the pregnancy within the first trimester (Table 2). Haemoglobin tests for anaemia had high coverage; out of the total number of registered pregnant women, there was > 100% coverage of testing more than four times, probably due to the discrepancy in service users compared to registered pregnant women (see Table 2). Coverage of two other key screening tests in pregnancy was lower. Less than one in 10 registered pregnant women were screened for gestational diabetes using an oral glucose tolerance test and 30.6% of women were tested for syphilis. There were gaps in treatment as well: 22.2% of women testing positive for syphilis received treatment and 40.2% of women diagnosed with severe anaemia did.

**Table 2:** Selected ANC services statistics from HMIS reported from public facilities for April 2019 to March 2020

	***Pregnant women registered for ANC (n* = *758,853)***^***1***^	
	n	%
***Contact***		
* Registered within first trimester*	541,828	71.4
* 4* + *ANC visits*	640,526	84.4
***Content***		
* Had ****blood sugar**** tested using OGTT*	64,899	8.6
* Had ****anaemia**** tested for 4* + *times*	834,292	109.9
* Of those tested, those diagnosed with severe anaemia*	52,632	6.3
* Of those with severe anaemia, those treated*	21,184	40.2
* Had ****syphilis**** test*	232,085	30.6
* Of those tested, those diagnosed with syphilis*	3,852	1.7
* Of those with syphilis, those treated*	857	22.2

^1^ Number of women registered for ANC at public sector facilities may not reflect the total number of service users, which can include pregnant women who registered before the stated period or who registered at one facility but sought care at multiple public and private facilities, sometimes in different districts, during their pregnancy. *ANC* = Antenatal Care,  *OGTT* =  Oral Glucose Tolerance Test

### Facility survey

**Fig. 2:**
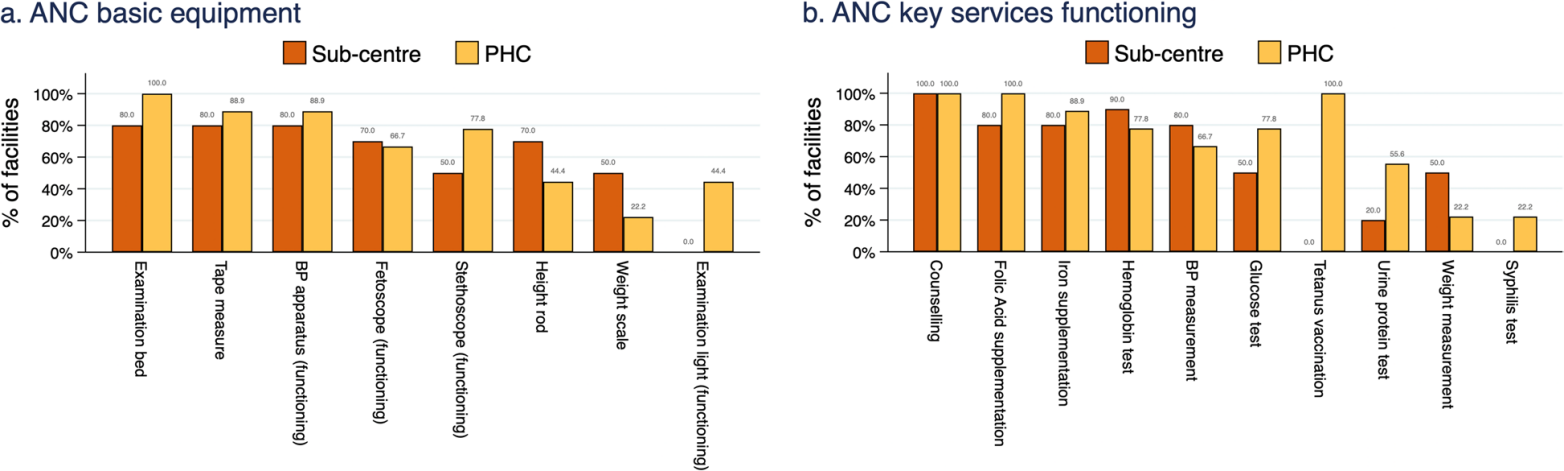
Percentage of facilities by level with available and functioning ANC basic equipment and offering ANC key services with corresponding functioning equipment and supplies

The mean scores for ANC basic equipment (out of a total of eight possible) were 4.8 and 5.3 at sub-centres and PHCs, respectively. Overall, only one facility surveyed (a PHC) had all eight items. The highest score in the sub-centres was observed to be seven, which was obtained in two of 10 facilities. The most frequently missed items of equipment in PHCs were a functioning examination light, weight scale and height rod (Fig. 2a). No sub-centres had a functioning examination light, and only half of sub-centres had a functioning stethoscope or weight scale.

The mean scores for ANC key services functioning (out of a total of 10 possible routine service components) were observed to be 5.5 and 7.1 for sub-centres and PHCs, respectively. Overall, no facilities surveyed offered all 10 components. The highest score observed in sub-centres was eight, seen in one of the 10 sub-centres surveyed, and the highest score observed in PHCs was nine, seen in two of the nine PHCs surveyed. Counselling on all eight core topics was reported provided in all the facilities. Tetanus vaccination and syphilis testing were not available at any of the sub-centres (Fig. 2b). Weight measurement and syphilis testing were available in two of the nine PHCs surveyed. Urine protein testing was available in five of the nine PHCs and in two of the 10 sub-centres surveyed.

### ANC observations

Of the 36 ANC observations, two were of women in their first trimester (≤ 12 weeks gestation), 14 in the second trimester (13 to 26 weeks gestation), and 20 in the third trimester (≥ 27 weeks gestation). Twenty-two observations were conducted with women of at least 22 weeks gestation.

For the respectful care domain (Supplementary Figure S1), a kind greeting was observed in 33 of the ANC consultations (91.7%) and the woman was offered a seat in 35 (97.2%). In only 22.2% of the ANC consultations were women asked if they had any other questions. A total of 35 women had a physical examination and, in only one of these, the healthcare provider discussed the steps of the physical exam with the woman. In none of the aforementioned 35 observations did the healthcare providers wash their hands before conducting the examination.

**Fig. 3:**
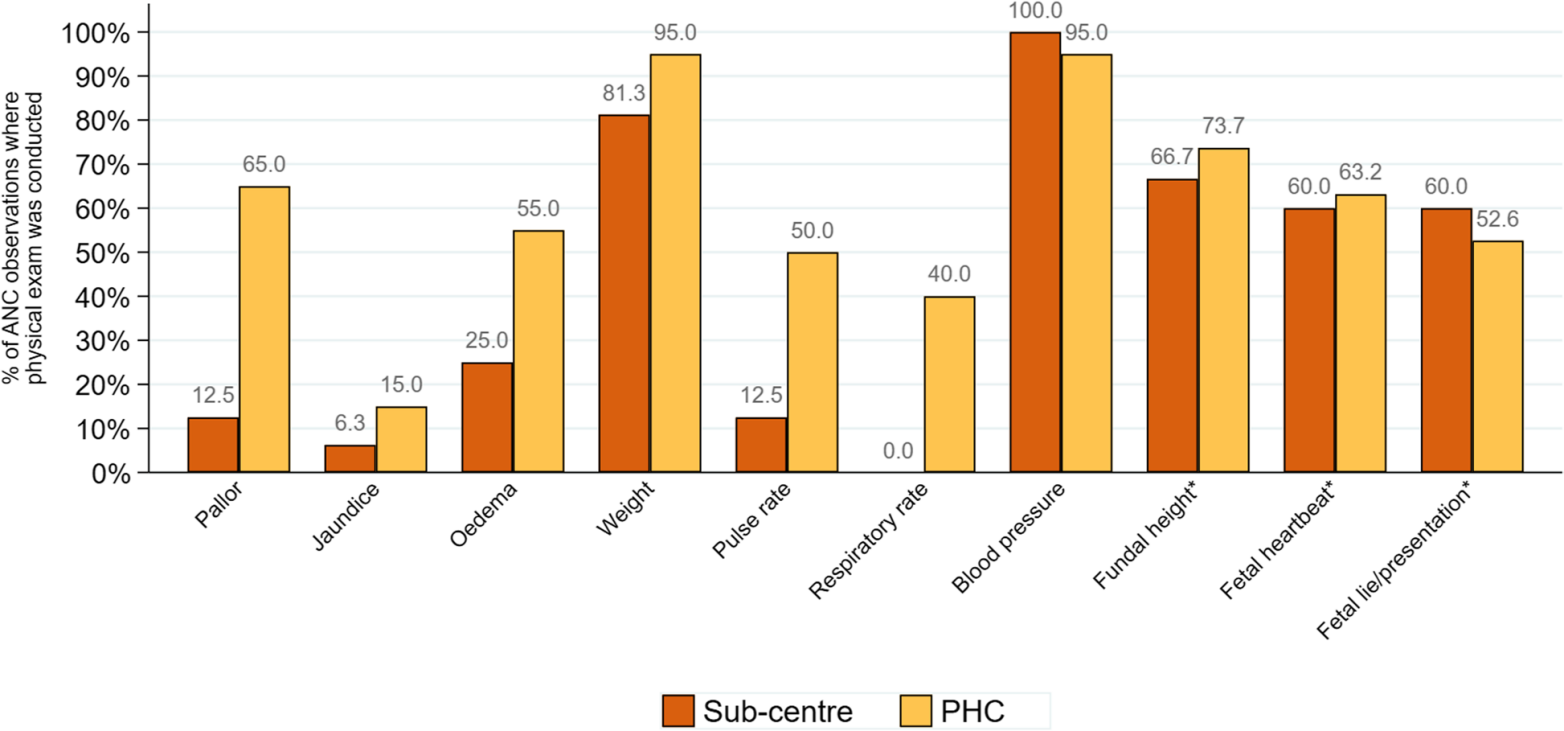
Percentage of ANC observations in which each of the 10 assessed physical examinations were conducted, stratified by facility type *Only includes ANC observations at 22 weeks gestation onwards (*n* = 22)

Figure 3 shows the percentage of ANC consultations in which each of the 10 assessed physical examinations were conducted. The highest number of physical examinations covered in a single ANC consultation were nine out of 10 (amongst three women). Overall, fewer than 50% of ANC observations had checks undertaken for oedema, pallor, pulse rate, respiratory rate and jaundice. Weight (88.9%) and BP (97.2%) were the two most frequently conducted physical examinations. However, there were some issues in the quality of the BP measurements. Amongst the nine indicators of good practice for measuring BP, no observations scored nine; however, six observations scored eight (Supplementary Table S1). Overall, 22.2% of women were tested, or referred for a test, for proteinuria, 69.4% for haemoglobin and 91.7% for blood/urine glucose. A total of 34 women (94.4%) were referred to another facility for syphilis testing.

Symptom assessment (Supplementary Figure S2) and education (Supplementary Figure S3) were generally done poorly. In very few observations were women asked about symptoms they had been experiencing, and the most commonly asked about symptom in the current pregnancy was decreased foetal movement (63.6%, 14/22 ANC observations among women of at least 22 weeks gestation). Only one woman was asked about her mental health. Nutrition and healthy eating were discussed in 77.8% of consultations, and the next ANC consultation details were discussed in 72.2%. Counselling of women on at least one of the ten items relating to birth preparedness and on at least one of six danger signs was done in 58.3% and 36.1% of consultations, respectively. Women were informed about pregnancy progress in 44.4% of consultations.

## Discussion

We analysed four data sources from Telangana that examined different aspects of quality of ANC, finding some important deficiencies in the quality of care despite high levels of utilisation. Analysis of the NFHS-5 for Telangana showed very high statewide coverage of assessed components, though counselling on pregnancy complications was the least performed component of care. Likewise, HMIS data showed high coverage of ANC visits but significant gaps in screening for syphilis and gestational diabetes. The facility survey in selected districts showed moderately equipped facilities. Some key services such as urine protein testing, which should be monitored regularly throughout pregnancy, and syphilis testing, which should be performed at least once during pregnancy, were unavailable in most of the facilities surveyed. While many ANC services or equipment items were commonly available individually, no facility in our sample offered all 10 components of routine ANC services. In the ANC observations, most women received adequate physical examinations, though some quality issues were noted in performance of BP measurement. However, symptom checking and client education were poorly done.

We found that clinical content of care, in particular maternal and foetal assessments, had high coverage and examinations – where we could evaluate these – were generally performed correctly. Despite high coverage of some important screening assessments (e.g., haemoglobin testing), there were also notable gaps in coverage and service availability (e.g., syphilis testing). This finding was obscured in the NFHS-5 results because women were only asked if they ever had their blood tested, not which specific tests performed. Some assessments that should be performed at every ANC visit, such as BP measurement, had nearly universal coverage in both the ANC observations and in the NFHS-5, which asked only if BP had been measured at least once during the pregnancy. However, other assessments, such as urine testing, had high coverage in the NFHS-5 when asked if urine testing had been performed at least once during the pregnancy, but coverage for urine protein testing, or referral for a test, showed considerable gaps in the ANC observations. This echoes findings from a survey of pregnant women in Kenya which found substantial disparities between receipt of key services at any point in pregnancy and receipt of those services at the recommended frequency [20].

We found that care often lacked the communication between the healthcare provider and pregnant woman that is important to high-quality, person-centred care [21]. The NFHS-5 and ANC observation data showed poor provision of information, with little counselling on potential signs of pregnancy complications. ANC observations showed poor psychosocial and emotional support. Few women in our ANC observations were asked about any current physiological symptoms or their mental health – important components of women’s experience of care [9]. Poor counselling in ANC has been documented in other LMIC settings, with calls for better measuring and improving the quality of information provision in ANC [22, 23]. The focus on guideline-driven care, particularly with increasing technical content of clinical care [10, 24] and emphasis on examinations, can negatively impact interpersonal aspects of quality [21]. Further, busy clinics or those with restricted hours or staff for ANC can often afford little time for meaningful provider-patient interaction [24].

Our findings demonstrate how data sources build upon or contradict one another to provide a fuller picture of the quality of ANC in Telangana, contributing to a growing body of literature on measurement of ANC quality [8, 9, 21, 25]. As others have found, components of ANC provision vary widely in quality and taking multiple data sources together can reveal quality gaps. For example, a study in rural Tanzania found that while pregnant women were highly satisfied by their care in exit interviews, data from observations and facility audits found ANC consultations frequently missed important care components, often due to stock-outs of medications and screening tests [26]. Another study of hospitals in Nepal found poor provision of recommended components during ANC observations; qualitative data from providers and pregnant women echoed these findings, attributing the observed poor performance to insufficient human resources, infrastructure and supplies [27]. Others have noted opportunities for integrating household survey and facility survey data to estimate composite measures of effective coverage of ANC interventions [28].

Indicators frequently drive the focus of improvement efforts [29]. Existing ANC quality measures mainly encompass indicators of content of care and of health system inputs with only a few measures of women's experience of care [8, 9]. Our ANC observation tool attempted to address this by examining whether women were asked about current pregnancy symptoms, given an explanation about the physical examination or given an opportunity to ask questions, drawing on components of respectful, person-centred ANC [20]. Given the historic relative emphasis on clinical assessments over counselling in ANC guidelines [10, 21], it is unsurprising that we found limited provision of information to pregnant women. Incorporating better measures of women’s experience of care will require greater consensus on what matters to women and what can be effectively measured, including through ANC observations, exit interviews with pregnant women, and household surveys [8, 9, 21].

Improving measurement of the quality of ANC includes opportunities to better assess responsiveness of care. For example, assessment of clinical practice could include whether women were told what their blood and urine samples were for and were given the results of screening tests. This could be assessed by observing ANC consultations or through exit interviews or surveys with pregnant women, although further validation work is needed on whether women can self-report this information. High-quality ANC should be responsive to individual women’s needs; where complications are identified, additional indicators on whether women received an appropriate response or treatment are needed.

Our analysis offers multiple strengths in bringing together four different data sources, but we encountered several limitations. Firstly, our data sources cover different time points, reducing some comparability of findings, particularly from the NFHS-5 five-year recall period. The facility survey and ANC observations were conducted in a small number of facilities in the selected five districts. While the facilities were randomly selected, the inclusion criteria for the sampling frame reflected logistical constraints and may not be representative of all PHCs and sub-centres in the districts.

Results from HMIS were hampered by questions about data quality and whether the available denominator – women registering their pregnancy at a public facility – was the most appropriate one. The counts of women extracted from the annual HMIS report (April 2019-March 2020) reflect imperfect numerators and denominators in a setting where pregnant women access care at many different facilities, including within different districts and within the public and private sector. So for example, while a pregnant woman might register at one public sector facility, and be recorded as receiving haemoglobin tests there, she might also receive multiple haemoglobin tests at different public facilities, leading to an overcounting of haemoglobin testing coverage as we observed. Despite this, the HMIS results yield a useful, though imperfect, picture of variability in service coverage.

The ANC observations offered invaluable insight into quality of care during a single ANC visit, but both data collection and analysis were challenging. We found it difficult to find the right balance between designing a data collection tool which covered all possible components of ANC and designing something which was feasible for fieldworkers to complete during the ANC observation. Pre-testing revealed the data collector would observe ANC consultations and later finish filling in the tool, as it was too challenging to observe and complete the long checklist. This introduced potential for misclassification or recall bias. Healthcare providers may also have improved the quality of care while under observation, though we note that substantial quality gaps remained. Additionally, analysing the observation data required integrating the results from the checklist with additional information about the woman’s stage of pregnancy and previous care received – elements that the tool was not fully designed to address. Each ANC observation was assessed individually by a clinically trained researcher, integrating information available in the woman’s handheld ANC card and whether or not it was the woman’s first ANC visit at that facility (or any facility). This limited the replicability of the analysis, and the amount of time needed to assess each ANC observation meant that this method would be challenging to do at large scale.

## Conclusion

The high coverage of contact with ANC services in Telangana and appearance of high-quality care as measured by receipt of selected care components obscured deficiencies in elements of quality. Some clinical assessments, such as BP measurement, showed consistently high coverage across multiple data sources, but important gaps around counselling, provision of information and psychosocial support remained. Household and facility survey and routine facility data are limited in capturing measures of a pregnant woman’s experience of care, but there may be scope for better capture of responsiveness of care provision and communication about the specific interventions and tests provided. Addressing these gaps will require indicators and data to measure progress towards achieving high quality in both content and experience of ANC.

## Supplementary Information

**Additional file 1:** **Supplementary Figure S1.** Percentage of ANC observations with components of respectful care, stratified by facility type. **SupplementaryTable S1.** Percentage of ANC consultations in which eachcomponent of measuring blood pressure correctly was observed (N=35, with oneANC observation excluded as there was no physical exam). **Supplementary Figure S2.** Percentage of ANC observations with in whichspecific symptoms were asked about in the ANC consultation, stratified byfacility type*Only includes ANCobservations at 22 weeks gestation onwards (N=22). **SupplementaryFigure S3.** Percentage of ANC observations with componentsof education, stratified by facility type.

## Data Availability

Data from the National Family Health Survey can be accessed from USAID’s Demographic and Health Survey program (https://dhsprogram.com/data/available-datasets.cfm). Health Management Information System data are available from the Ministry of Health & Family Welfare, India. De-identified data from the facility survey and antenatal care observations can be requested from Dr. Monica Chaudhry (monica.chaudhry@phfi.org).

## Abbreviations

ANC: Antenatal care
BP: Blood pressure
CHFW: Commissionerate of Health and Family Welfare
Hb: Haemoglobin
HMIS: Health management information system
LMIC: Low- and middle-income country
MoHFW: Ministry of Health and Family Welfare
NFHS-5: National Family Health Survey, 2019–21
OGTT: Oral glucose tolerance test
PHC: Primary health centre
WHO: World Health Organization
