# Supplementary material for: Measuring the quality of antenatal care in a context of high utilisation: evidence from Telangana, India
Source: BMC Pregnancy Childbirth. 2022 Nov 25;22:876. doi: 10.1186/s12884-022-05200-1 (PMC9700993; doi:10.1186/s12884-022-05200-1)
Supplement: Supplementary file 1 — Additional file 1: Supplementary Figure S1. Percentage of ANC observations with components of respectful care, stratified by facility type. SupplementaryTable S1. Percentage of ANC consultations in which eachcomponent of measuring blood pressure correctly was observed (N=35, with oneANC observation excluded as there was no physical exam). Supplementary Figure S2. Percentage of ANC observations with in whichspecific symptoms were asked about in the ANC consultation, stratified byfacility type*Only includes ANCobservations at 22 weeks gestation onwards (N=22). SupplementaryFigure S3. Percentage of ANC observations with componentsof education, stratified by facility type. [file 12884_2022_5200_MOESM1_ESM.docx]

# Supplementary Material


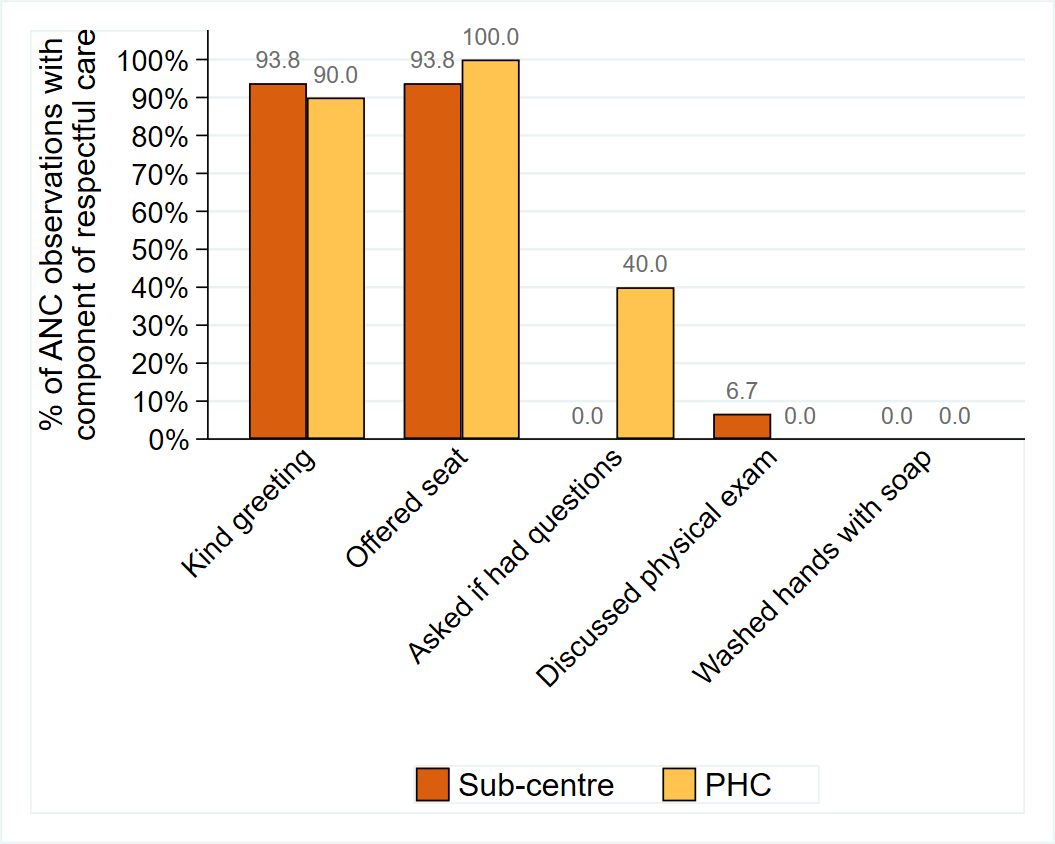


Supplementary Figure S1: Percentage of ANC observations with components of respectful care, stratified by facility type

Supplementary Table S1: Percentage of ANC consultations in which each component of measuring blood pressure correctly was observed (N=35, with one ANC observation excluded as there was no physical exam)

| **Blood pressure component** | **Percentage with component of blood pressure measured correctly** | |
| --- | --- | --- |
|  | **Sub-centre (n=16)** | **PHC**  **(n=19)** |
| Asked if the patient had tea or coffee | 31.3 | 52.6 |
| Back supported during BP measurement | 12.5 | 15.8 |
| Feet rested | 87.5 | 94.7 |
| Measurement taken on the left arm | 81.3 | 52.6 |
| Arm rested on the table | 56.3 | 79.0 |
| Asked to roll-up sleeve | 81.3 | 73.7 |
| Lower band of cuff positioned 1-2cm above the elbow | 87.5 | 84.2 |
| Cuff at heart level | 87.5 | 94.7 |
| Deflation rate of not more than 2 to 3 mm Hg/s | 100 | 89.5 |


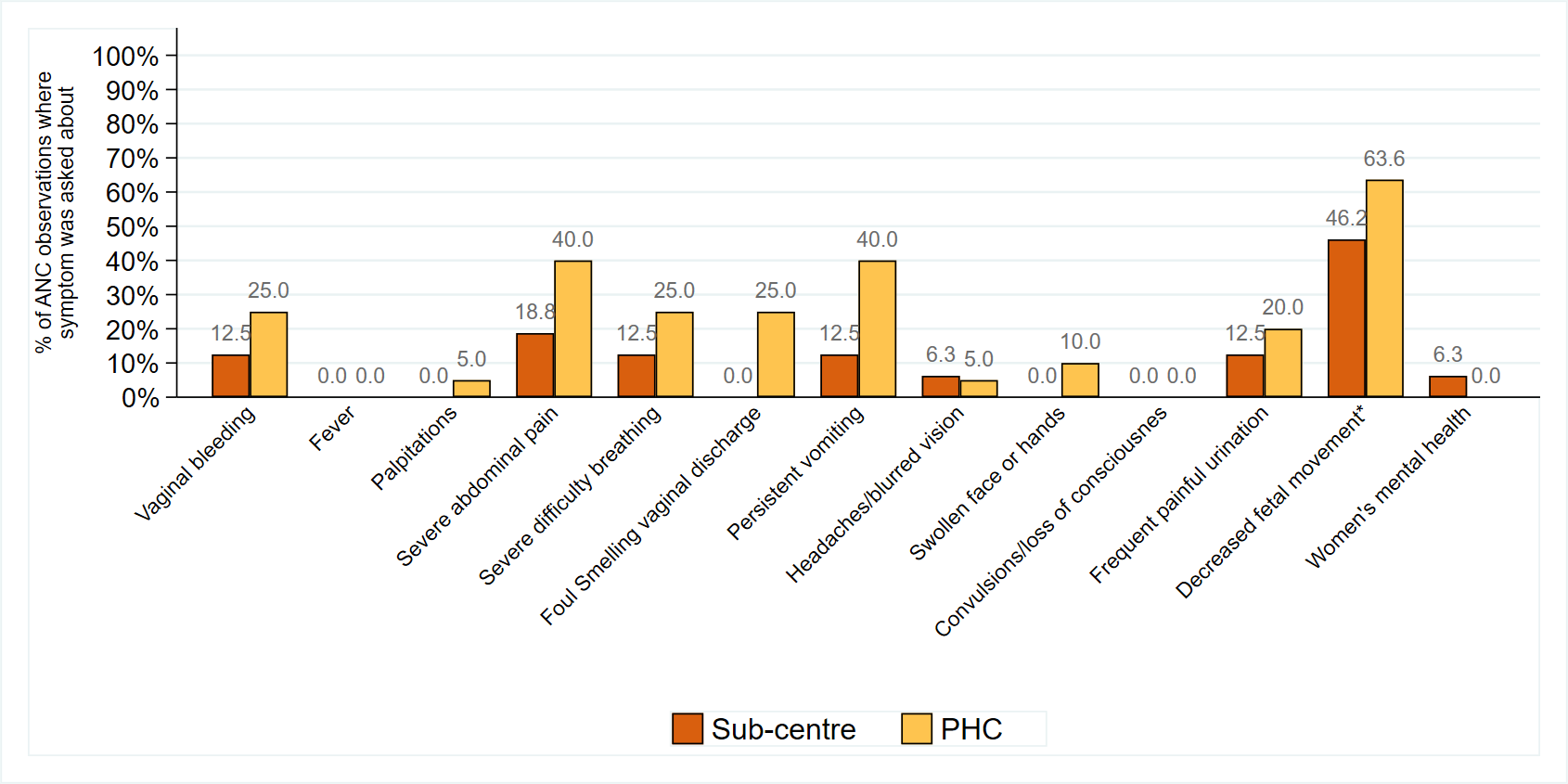


Supplementary Figure S2: Percentage of ANC observations with in which specific symptoms were asked about in the ANC consultation, stratified by facility type

*Only includes ANC observations at 22 weeks gestation onwards (N=22)


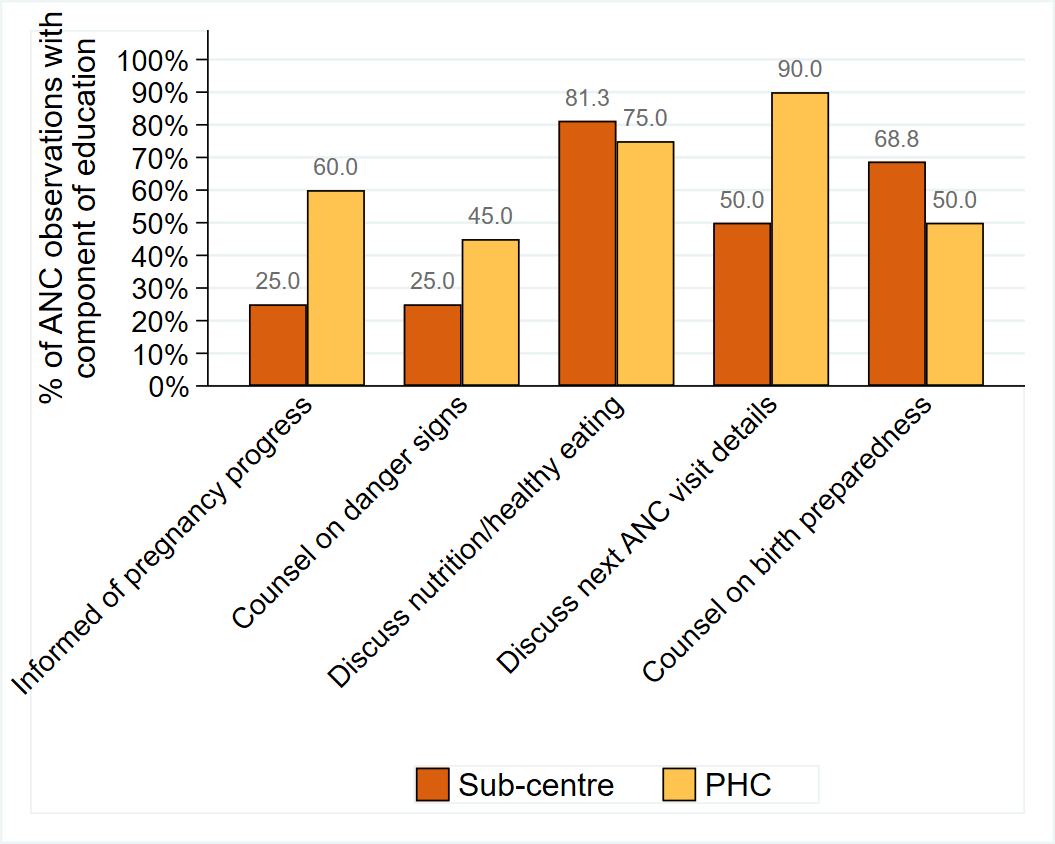


Supplementary Figure S3: Percentage of ANC observations with components of education, stratified by facility type
